# Synergistic Activation of Latent HIV-1 Expression by Novel Histone Deacetylase Inhibitors and Bryostatin-1

**DOI:** 10.1038/srep16445

**Published:** 2015-11-13

**Authors:** Marta Martínez-Bonet, Maria Isabel Clemente, Maria Jesús Serramía, Eduardo Muñoz, Santiago Moreno, Maria Ángeles Muñoz-Fernández

**Affiliations:** 1 Laboratorio de InmunoBiología Molecular, Hospital General Universitario Gregorio Marañón; Spanish HIV BioBank, Instituto de Investigación Sanitaria Gregorio Marañón (IISGM), C/Dr. Esquerdo 46, 28007 Madrid, Spain; 2 Networking Research Center on Bioengineering, Biomaterials and Nanomedicine (CIBER-BBN); C/Dr. Esquerdo 46, 28007 Madrid, Spain; 3 Unidad de cultivos celulares, Instituto de Investigación Sanitaria Gregorio Marañón (IISGM), C/Dr. Esquerdo 46, 28007 Madrid, Spain; 4 Department of Cell Biology, Physiology and Immunology, Instituto Maimónides de Investigaciones Biomédicas de Córdoba (IMIBIC)/Reina Sofia University Hospital/University of Córdoba, Córdoba, Spain; 5 Servicio de Enfermedades Infecciosas, Hospital Universitario Ramón y Cajal and IRYCIS, Madrid, Spain

## Abstract

Viral reactivation from latently infected cells has become a promising therapeutic approach to eradicate HIV. Due to the complexity of the viral latency, combinations of efficient and available drugs targeting different pathways of latency are needed. In this work, we evaluated the effect of various combinations of bryostatin-1 (BRY) and novel histone deacetylase inhibitors (HDACIs) on HIV-reactivation and on cellular phenotype. The lymphocyte (J89GFP) or monocyte/macrophage (THP89GFP) latently infected cell lines were treated with BRY, panobinostat (PNB) and romidepsin (RMD) either alone or in combination. Thus, the effect on the viral reactivation was evaluated. We calculated the combination index for each drug combination; the BRY/HDACIs showed a synergistic HIV-reactivation profile in the majority of the combinations tested, whereas non-synergistic effects were observed when PNB was mixed with RMD. Indeed, the 75% effective concentrations of BRY, PNB and RMD were reduced in these combinations. Moreover, primary CD4 T cells treated with such drug combinations presented similar activation and proliferation profiles in comparison with single drug treated cells. Summing up, combinations between BRY, PNB and/or RMD presented a synergistic profile by inducing virus expression in HIV-latently infected cells, rendering these combinations an attractive novel and safe option for future clinical trials.

Effective combination antiretroviral therapy (cART) has improved the quality of life and the life expectancy of HIV-infected patients. Nevertheless, achieving the cure of HIV is still an unattainable challenge for the scientific community. Although cART achieves undetectable plasma viral RNA and the normalization of CD4 T cell levels in the majority of patients, several studies have shown that HIV remains incurable owing to the persistence of latently infected cells^1^,^2^,^3^,^4^. Most of these cells are resting memory or naïve CD4 T cells and other cells belonging to the monocyte/macrophage lineage that contain integrated provirus within their genome^5^,^6^. This latent infection escapes from the cART effect and remains undetectable to the immune system.

Several therapeutic interventions to eradicate HIV focus on the stimulation of viral production from latently infected cells. This is followed by a “kill” phase that permits the elimination of infected cells through existing immune responses or cytotoxic drugs under the aim to purge and clear HIV reservoirs. This strategy involves the use of a wide range of small molecules called latency-reversing agents (LRAs)^7^. Such drugs include: (1) histone deacetylase inhibitors (HDACIs)^8^,^9^ (2) disulfiram, postulated to involve the nuclear factor κB (NF-κB) activation^10^,^11^ (3) bromodomain-containing protein 4 (BRD4) inhibitor JQ1, which elicits effects through positive transcription elongation factor (P-TEFb)^12^ and (4) protein kinase C (PKC) activators such as ingenols^13^, prostratin^14^, 1,2-diacylglycerol analogues^15^ and bryostatin-1 (BRY)^16^,^17^. Not only the interest in these drugs has grown greatly, but also the number of on-going clinical trials about the safety and the effect of LRAs as disruptors of HIV latency have increased. HDACIs are the most advanced HIV-1 anti-latency agents in current clinical testing, mainly due to the synthesis in recent years of novel and more specific pan-HDACIs, such as givinostat, belinostat and panobinostat (PNB)^18^,^19^ and newly synthesized class I selective HDACIs that include oxamflatin^20^, NCH-51^21^ and romidepsin (RMD)^22^. Recently, published results validate the safety and the effect of PNB on HIV expression in patients on suppressive cART in a clinical trial, and postulated this compound as a promising reactivator of HIV viral latency. However, this study reveals that PNB did not reduce the number of latently infected cells and must be combined with other drugs in order to significantly affect the size of HIV reservoirs^23^. Consistent with these results Bullen, C. K., *et al.* have shown a comparative *ex vivo* evaluation of different LRAs demonstrating that none of the leading candidate drugs can singly disrupt the latent HIV reservoir. Thereby, the combination of several LRAs can be the best strategy for HIV latency reactivation and precludes possible synergisms^24^. Indeed, some published results described possible synergisms between the classic HDACIs (valproic acid, vorinostat and sodium butyrate) and either prostratin^25^ or BRY^26^ in HIV expression activation, possibly due to the other role attributed to HDACIs as *stimuli* promoting NF-κB activity^27^. Many other combinatorial approaches have been postulated as good strategies to induce the reactivation of latent reservoirs^24^,^28^,^29^. Therefore, we study the possible synergism between the new promising HDACIs PNB or RMD and BRY as non-carcinogenic PKC activator, in order to reveal new insights in LRA combinatorial strategies that could be useful for future clinical trials design.

## Results

### Enhanced reactivation profile of bryostatin-1 and HDACIs combinations in latently HIV-1 infected cells

**Figure 1:**
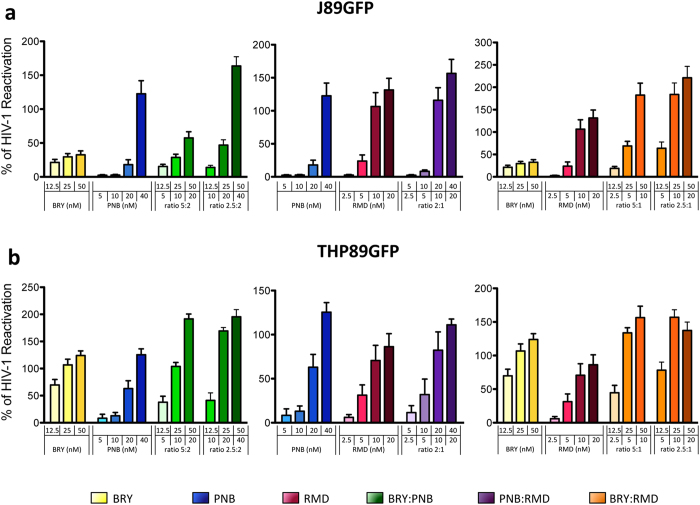
Reactivation-effect of drugs alone or in double combinations. J89GFP **(a)** and THP89GFP **(b)** cell lines were treated with BRY (yellow), PNB (blue) and RMD (red) at the indicated concentrations, alone or in the following double combinations: BRY:PNB (green), PNB:RMD (purple) and BRY:RMD (orange) at the specified ratios. After 24 hours, HIV reactivation was analysed by flow cytometry as EGFP expression (iMFI). Percentage of HIV reactivation was normalized to TNF-induced viral reactivation. Results represent the arithmetic mean + SEM of at least three independent experiments.

As an experimentally tractable and relevant model to study post-integration HIV latency and reactivation^30^, we employed J89GFP and THP89GFP, which are respectively lymphocyte and monocyte-derived cell lines latently infected by HIV that carry out a copy of latent EGFP under the control of HIV promoter. The HIV reactivation effect of BRY, PNB and RMD was tested as individual drugs or in combination at different ratios. The election of the ratios was performed taking into account the non-toxic concentration for each drug (BRY: 12.5–50 nM, PNB: 5–40 nM, and RMD: 2.5–20 nM) (see Supplementary Figs S1 and S2 online). After 1 day of exposure, the reactivation effect was measured by flow cytometry and expressed as EGFP (integrated MFI or iMFI, see Supplementary Fig. S3 online). Moreover, flow-cytometry profiles of a representative experiment are depicted in Supplementary Fig. S4 online, showing both cell viability and HIV-reactivation by 7AAD staining and EGFP detection, respectively. Nevertheless, to avoid experimental deviations, results were normalized according to the EGFP expression profile of TNF-α treated cells (Fig. 1).

To research the reactivation activity of each individual drug in these HIV latency models, we analysed the dose-effect curves in the two cell lines (see Supplementary Fig. S5 online), and we determined the effective concentration (EC_50_, EC_75_, EC_90_, and EC_95_) of each drug (Table 1). The effective concentration of the drugs in J89GFP cells was higher than in THP89GFP and BRY. The doses needed to obtain an effective reactivation in J89GFP cells were extremely high (micromolar range), exceeding the commonly used nanomolar range. As shown in Fig. 1 and Supplementary Fig. S3 online, all of the tested combinations containing BRY and HDACIs resulted in a more efficient reactivation profile in either J89GFP and THP89GFP cell lines compared to the effects of the individual drugs. On the contrary, the combination of PNB and RMD, slightly improved the single drug effect.

To evaluate the type of drug interactions, we calculated the combination index (CI) values (Table 2). For this purpose, the CalcuSyn software based on the median effect principle was used^31^,^32^. The obtained CI values demonstrated the positive interactions between BRY and HDACIs, and synergy was observed for the majority of the BRY+PNB and BRY+RMD combinations in both HIV latently infected cell lines. The only exception was the EC_50_ values in THP89GFP cell line, which showed non-synergism. Nevertheless, very strong or strong synergistic interactions were observed at higher effective doses. On the other hand, non-synergistic or additive effects were observed when PNB was mixed with RMD. Since enhanced reactivation effects and generally synergistic profiles were observed when BRY was combined with the HDACIs, the use of both HDACIs must be challenged for future utilization. Consequently, even though slight or no synergism was observed between PNB and RMD, we wondered whether the triple combination BRY+PNB+RMD could render a positive interaction. The ratios assessed in the double combinations were maintained, resulting in two different triple combination ratios 2.5:2:1 and 5:2:1. As depicted in Table 3, a potent synergistic profile (CI values between 0.22–0.76) was found at calculated EC_50_, EC_75_, EC_90_, and EC_95_ for both ratios in J89GFP cells, whereas a moderate to strong synergistic effect (CI values between 0.4–0.84) tended to appear only at the highest calculated effective doses (EC_90_, and EC_95_) in THP89GFP cells. Summing up, we demonstrated that BRY is highly efficient in combination with HDACIs and that PNB and RMD can be used efficiently when mixed with BRY to reactivate HIV from latency in our cell line models.

### Reduction of effective concentration values when bryostatin-1 and HDACIs were used in double or triple combinations

**Figure 2:**
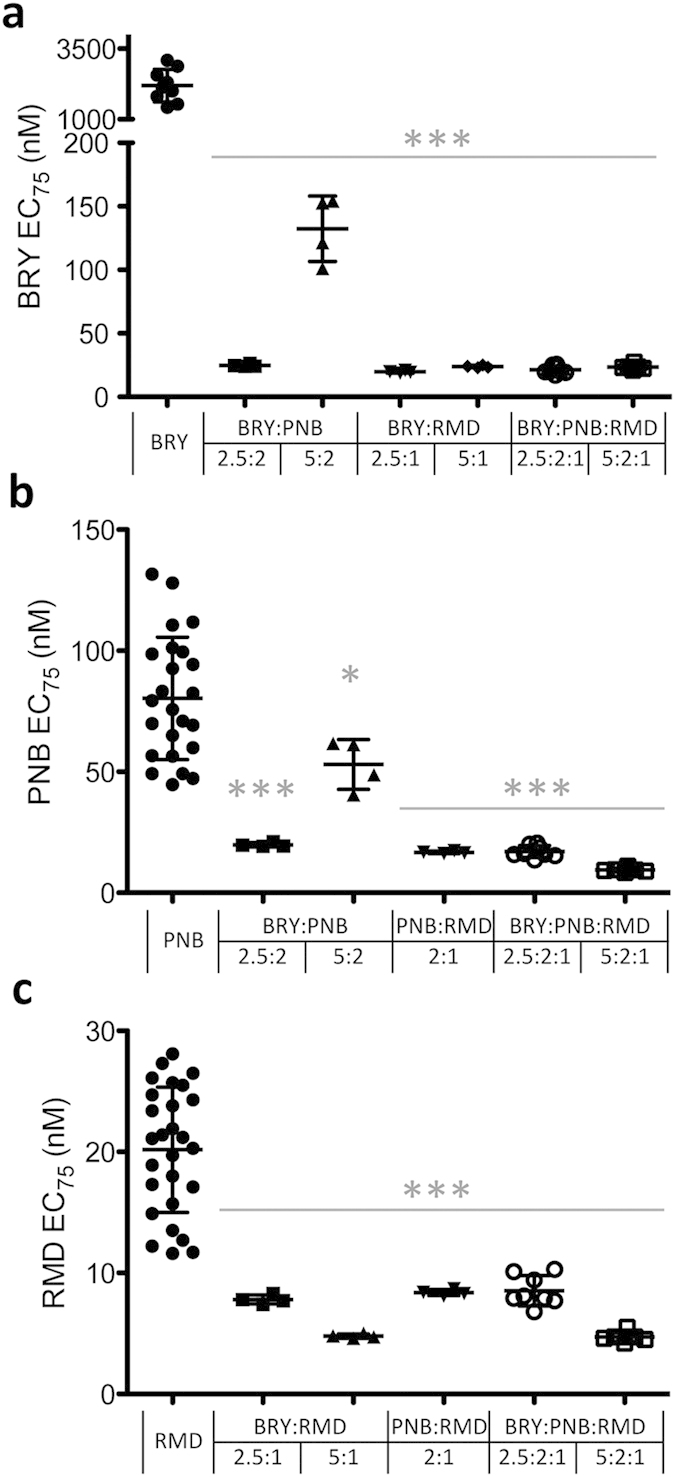
Dose-effect EC_75_ values of drugs alone or in combinations in J89GFP cells. The 75% effective reactivation concentration (EC_75_) values (nM ± SD) in latently infected J89GFP cells of BRY **(a)**, PNB **(b)** and RMD **(c)** drugs alone and in combination at the indicated ratios 24 hours post-treatment are shown. Each symbol represents results from of at least three independent experiments and the mean ± SD is shown. Statistics were performed between the calculated EC_75_ values of the drug at single and combined treatments. ***p < 0.001; *p < 0.05.

Once demonstrated the synergistic effect of BRY/HDACIs combinations, we analysed their implications in terms of effective concentration. Based on the *in vitro* results, the CalcuSyn software permits to determine the dose-reduction-index of each drug^32^,^33^. Therefore, the effective concentration of each single drug when combined with others can be predicted. We first analysed the reactivation effect of the two-drug combinations containing BRY+PNB, BRY+RMD and PNB+RMD. All the evaluated combinations achieved a statistically significant reduction in the EC_75_ values, as compared with the EC_75_ values for each individual drug, in both J89GFP (Fig. 2) and THP89GFP (Table 4) cell lines. For example, BRY alone reactivated HIV expression in J89GFP cells with an average EC_75_ of 2,020 nM (Fig. 2), meanwhile the combination of BRY and PNB (ratio 2.5:2) or RMD (ratio 2.5:1) reactivated latent HIV with an average EC_75_ of 24.81 or 19.53 nM, respectively. Therefore, to achieve the same level of HIV reactivation, 80- and 100-fold less concentration of BRY in combination with PNB and RMD respectively was needed, when comparing the effect of BRY as a single drug. The EC_75_ of PNB and RMD were also reduced in these combinations, showing an average of 4-fold decrease in the concentrations used. In addition, the EC_75_ of each drug in the triple combination BRY:PNB:RMD (at 5:2:1 ratio), reached very similar EC_75_ average values in both cell lines: 23.57 nM, 9.45 nM and 4.72 nM (J89GFP) and 20.68 nM, 8.27 nM and 4.14 nM (THP89GFP) for BRY, PNB and RMD, respectively (Fig. 2 and Table 4).

### Effect of BRY, PNB and/or RMD combinations on primary human T cells phenotype

**Figure 3:**
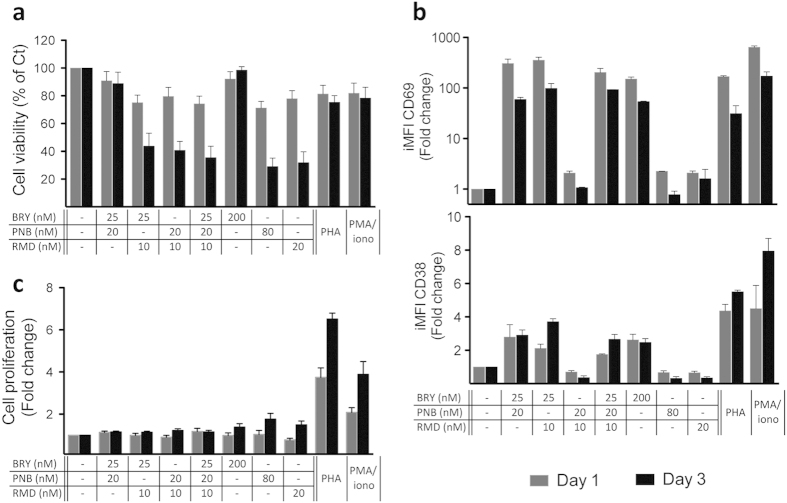
Primary human cells phenotype after treatment with selected drug combinations. Purified CD4 T cells from healthy subjects were treated with the indicated concentrations of BRY, PNB, RMD or with PHA or PMA/ionomycin for 1 (grey bars) and 3 (black bars) days. (**a**) Cell viability was determined using 7AAD reagent and analysed by flow cytometry and expressed as percentage. (**b**) The surface expression of the activation markers CD38 and CD69 in viable CD4 T cells were analysed by flow cytometry and expressed as iMFI. (**c**) Cell proliferation was determined as BrdU incorporation measured by ELISA. Results are normalized to control vehicle-treated CD4 T cells from the same donors and represent the mean + SEM of three independent experiments.

BRY has been previously reported to be potentially associated with uncontrolled T cell activation at concentrations required for efficient HIV reactivation. In this study, we described that BRY, PNB and/or RMD combinations showed a strong synergism in the reversion of latency in J89GFP cells (Tables 2 and 3) and that the EC values of each drug diminished significantly in the combinations in comparison with single drug treatment (Fig. 2). We analysed if the lower EC_75_ doses achieved in these LRA combinations could improve the effect of single drug treatment at their EC_75_ in terms of viability, activation and proliferation profile in primary T cells. We selected the combination ratio 2.5:2:1 (BRY:PNB:RMD), since it achieves lower BRY EC_75_ values, approximately 25 nM in J89GFP cell line (Fig. 2). Isolated primary human CD4 T cells were treated with the double or triple combinations (BRY 25 nM, PNB 20 nM and/or RMD 10 nM) or with the single drugs at their calculated EC_75_ values (PNB 80 nM, RMD 20 nM) or with the maximal non-toxic concentration (BRY 200 nM, ten-fold below of its EC_75_ dose). PHA or PMA/ionomycin, which are *stimuli* that uniformly reactivate latent HIV in almost all the systems previously tested were used as controls^24^,^34^. Either one or three days after CD4 T cell treatment, we determined the cell viability and the activation profile by flow cytometry. Cell proliferation was measured by ELISA by BrdU incorporation. Cell death was analysed by 7AAD incorporation by flow cytometry (Fig. 3a). A good cell viability was observed one day post-treatment, whereas it was compromised at day three in BRY+RMD, PNB+RMD and BRY+PNB+RMD combinations, probably due to the effect of RMD, plausible since the highest concentration of BRY or the combination BRY+PNB presented values over 80% of cell viability.

The effects on T cell activation after BRY, PNB and/or RMD exposure were analysed by measuring the iMFI of CD38 and CD69 activation markers in living cells (Fig. 3b). As expected, CD69 expression was markedly increased and sustained over three days, in CD4 T cells treated with BRY, either alone or in combination with PNB and/or RMD. It is well known that BRY activates NF-κB pathway and that CD69 gene presents three NF-κB binding sites in its promoter region^35^. On the other hand, CD38 expression was slightly increased in CD4 T cells treated with BRY, either alone or in combination with PNB and/or RMD, but presented levels between 2 and 4 times lower compared to the conventional PHA or PMA/ionomycin treatments.

Representative flow cytometry profiles of CD4 T cell viability and activation markers expression are shown in Supplementary Fig. S6 online.

Finally, cell proliferation was measured on CD4 T cells. Figure 3c shows that the treatment with the LRAs at the maximum concentrations tested produced a 1.5 to 2-fold increase in cell proliferation after three days of treatment, values consistently lower than those obtained after PHA or PMA/ionomycin treatment. Furthermore, the double or triple BRY, PNB and/or RMD combinations diminished the effect of single drugs, presenting proliferation values of non-treated cells.

## Discussion

The persistence of HIV-1 involves numerous overlapping cellular pathways, which are interesting from the pharmacological point of view. Thus, targeting multiple steps within the virus latency mechanisms will be important to optimize the reactivation effect and hence permitting the elimination of HIV reservoirs trace.

In a recent study exploring a panel of HDACIs for HIV reactivation, were an *in vitro* latency assay was used, PNB displayed superior potency to multiple other HDACIs tested including givinostat, belinostat and vorinostat^28^. Moreover, has already been described^33^ the great ability of RMD to reverse HIV latency *in vitro* and *ex vivo* in resting and memory CD4 T cells from HIV-infected patients on suppressive cART, in comparison with vorinostat and other HDACIs currently in clinical development. BRY has also be remarked as the best effective single agent between a battery of LRAs that disrupt the latent reservoir^24^. Furthermore, the synergy between prostratin^25^ or BRY^26^ and classical HDACIs has been previously described. Nevertheless, the treatment with HDACIs has been shown to mobilize the latent reservoir but could have unintended negative impacts on the effector functions of CTL, thus impairing elimination of infected cells^36^,^37^. In addition, the administration of PNB and BRY to patients has been questioned due to significant toxicity levels observed in cell lines and resting CD4 T cells^38^. Finally, low concentrations of PKC agonists regulate a different set of genes compared to high concentrations of these compounds, which may have clinical implications in terms of potential side effects mediated by the activation of the NF-κB canonical pathway (Muñoz *et al.*, *manuscript in preparation*). Therefore, a combinatorial strategy can lead to a reduction in the concentration of LRAs used *in vivo*, resulting in a reduction of adverse effects, limiting the local injuries, the toxicity and the inflammation.

As mentioned above, PNB, RMD and BRY presented a good reactivation activity both *in vitro* and *ex vivo* and are currently used in HIV eradication clinical trials^23^,^39^,^40^. To attack HIV latency through HDAC inhibition and NF-κB activation either at epigenetic and transcriptional levels respectively, we studied the possible synergism between these compounds. Such study could bring new insights about the possibility of reactivating virus transcription either with high efficiency and the lowest concentration possible in order to reduce their potential toxicities. For this purpose, we have employed J89GFP and THP89GFP cell lines to explore the impact of PNB and/or RMD in combination with BRY. Although J89GFP and THP89GFP cell lines are not frequently used to assess LRAs^41^,^42^,^43^,^44^,^45^,^46^,^47^, their exclusive characteristics make from them an experimentally tractable and relevant model to study post-integration HIV latency and reactivation^30^. Nevertheless, to confirm the results obtained in these cell lines, we have performed a reactivation experiment with BRY, PNB, RMD and the drug combinations selected for primary cells treatment in another two different well- established latency-models, such as ACH-2 and J1.1 cell lines. The obtained data are in concordance to those exposed in this article and are shown in Supplementary Figs S7 and S8 online, which represent cell toxicity (measured by MTT assay) and HIV reactivation (Ag p24 in cell supernatants measured by ELISA), respectively.

All of the tested combinations containing BRY and HDACIs resulted in a more efficient reactivation profile in both the J89GFP and THP89GFP cell lines compared to the effects of the individual drugs. Indeed, the obtained CI values demonstrated synergism between BRY and HDACIs in both HIV latently infected cell lines, whereas slight or no synergism was observed between PNB and RMD. Since both drugs target HDAC, their use in combination could be inducing a competition between them, resulting in an inefficient anti-latency mechanism. On the other hand, the statistically significant reduction in the effective dose values of drugs in combination, as compared to each individual drug, suggested that this kind of combinatorial approach could achieve a strong mobilization of latent reservoirs while diminish side effects.

CD4 T cells are one of the main subsets of cells in which HIV latency is established, and the activation state of this cell type is pivotal for active HIV production. Therefore, the level of phenotype changes in primary human CD4 T cells was assessed after BRY, PNB and/or RMD treatment. We did comparisons between the EC_75_ doses achieved in drug combinations and the EC_75_ of single drugs. High cell viability was observed at day one post-treatment, whereas it was compromised at day three in BRY+RMD, PNB+RMD and BRY+PNB+RMD combinations. Since PNB induces prolonged histone hyper acetylation^48^, pulses instead of continuous treatment or intermittent dosing schedules should be evaluated. In terms of cell activation, we found that none of the combinations augmented the activation effect of the single drugs at their EC_75_ values, and that double or triple BRY, PNB and/or RMD combinations presented similar proliferation values to non-treated cells, diminishing the effect of single drugs at their EC_75_ value.

Although administered within the context of cART, the infection of bystander cells remains a concern in a “shock and kill” therapeutic approach. Both HDACIs and BRY may have a negative impact in this aspect, due to their effect on CTL impairment or T cell activation, respectively. On the contrary, these compounds also have a positive impact on HIV inhibition: among other HDACIs, PNB and RMD enable the decrease of HIV release from macrophages via the degradation of intracellular HIV through the canonical autophagy pathway^49^ and BRY is able to inhibit acute HIV infection by diminishing surface expression of receptors and co-receptors or in a receptor independent manner^16^.

We are aware that the main limitation of this study is the use of an *in vitro* cell latency model. The rarity of resting CD4 T cells latently infected makes this type of study challenging. Moreover, it has been remarked the discordance between the effects of non-stimulating LRAs in *in vitro* models of HIV latency and their effects in *ex vivo* in resting CD4 T cells from infected individuals on cART. This data indicates that these models do not fully capture all the mechanisms governing HIV latency *in vivo*. In fact, based on the screening depicted in this article, further efforts should be carried out in *ex vivo* models in order to find the most appropriate concentrations of these combinations, particularly before any further *in vivo* studies. On the other hand, the assays presented herein facilitated that is to our knowledge the first *in vitro* quantitative synergistic evaluation of candidate LRAs administered in combination in both lymphocyte and monocyte/macrophage HIV latently infected lineages.

## Methods

### Reagents

PE-conjugated anti-human CD69 and FITC-conjugated anti-human CD38 were obtained from BD Biosciences Pharmigen (San Diego, CA, USA). BRY was obtained from Sigma-Aldrich (St. Louis, MO, USA), PNB and RMD were obtained from Selleck Chemicals (Houston, TX). Drugs were dissolved in dimethyl sulfoxide (DMSO) (Sigma-Aldrich, St. Louis, MO, USA) to prepare stock solutions. DMSO concentration in cell cultures was lower than 0.001%. TNF-α was obtained from R&D Systems (Minneapolis, Minn.). PhytohemaGglutinin (PHA), phorbol 12-myristate 13-acetate (PMA) and ionomycin were purchased from Sigma-Aldrich.

### Cell lines and culture

J89GFP and THP89GFP cell lines (kindly donated by Dr. David N Levy, NYU, USA) were maintained according to the protocol described in^30^. ACH-2 and J1.1 cells were obtained through the NIH AIDS Reagent Program, Division of AIDS, NIAID, NIH from Dr. Thomas Folks^45^,^50^,^51^. Buffy coats from healthy subjects were obtained from the Madrid Transfusion Center. CD4 T cells were purified by negative immunomagnetic separation (CD4+ T Cell Isolation Kit; Miltenyi Biotech, Friedrich, Germany) from peripheral blood mononuclear cells (PBMCs) isolated from heparinised venous blood by fycoll-paque density gradient and maintained in complete RPMI supplemented with 30 U/ml IL2 (Murex Biotech, England, UK)^52^.

### Cell viability assays

The concentration range of each compound assayed in this study is in agreement with previously published results^26^,^28^. The toxicity of compounds was measured by MTT (Sigma-Aldrich) assay according to the manufacturer’s instructions in J89GFP and THP89GFP cell lines. 0.001% DMSO treated cells were included in each experiment as vehicle control (Ct); DMSO (10%) was used as positive control of cytotoxicity. Cell death in J89GFP, THP89GFP and primary human CD4 T cells was also determined by 7-aminoactinomicyn-D (7AAD) intercalation and analysed in a Gallios flow-cytometer (Beckman-Coulter, CA, USA). Results were expressed as row data or normalized to control vehicle-treated cells as depicted in each figure.

### Analysis of cell activation profile

Cells were stained for 30 minutes at 4°C with the corresponding conjugated antibodies in FACS staining buffer (phosphate-buffered saline (PBS) with 2% FBS), and surface activation markers expression was analysed in live cells in a Gallios flow-cytometer (Beckman-Coulter). At least 50,000 CD4 T cells were collected for each sample and analysed with Kaluza software (Beckman-Coulter). Results were normalized to control vehicle-treated CD4 T cells from the same donors.

### Latent HIV-1 reactivation

EGFP-fluorescence pattern measured by flow cytometry was used to determine viral reactivation in J89GFP and THP89GFP cell lines. Whereas Agp24 release measured by enzyme immunosorbent assay (ELISA, INNOTEST® HIV-Antigen mAb, Innogenetics, Belgium) was used to determine viral reactivation in ACH-2 and J1.1 cells. J89GFP and THP89GFP cells were stimulated with the indicated compounds and stained with 7AAD to assess cell viability. At least 30,000 cells were analysed by flow cytometry. The integrated mean fluorescence intensity (iMFI, percentage of EGFP expressing cells ^*^MFI) of live cells was used as a measure of HIV reactivation and standardized with the reactivation obtained after TNF-α treatment. The 50%, 75%, 90% and 95% effective concentrations (EC_50_, EC_75_, EC_90_ and EC_95_, respectively) were determined, and synergism analysis was performed using the CalcuSyn software (Biosoft, Cambridge, UK), based on the median effect principle^33^. The combination index (CI) of each drug combination was plotted as a function of the fractional inhibition by computer simulation; the fractional inhibition values ranged from 0.10–0.95. CI values between 0.1–0.9 indicate a synergistic effect; whereas, values between 0.9–1.1 represent an additive effect, and >1.1 represents antagonism. Each experiment was performed in triplicate.

### Cell proliferation assays

CD4 T cell proliferation was assayed by using the bromodeoxyuridin (BrdU) Cell Proliferation Kit (Chemicon, Millipore, MA, USA) according to the manufacturer’s instructions. PHA (2 μg/ml) or PMA (2.5 ng/ml) plus ionomycin (250 ng/ml), which are *stimuli* that uniformly reactivate latent HIV in almost all the systems previously tested^24^,^34^ were used as controls. Cell proliferation assays were performed in triplicate.

### Statistics

Statistical analysis was performed using SpSS software version 15.0 for Windows. Differences between two groups (control versus different dosages of compounds or BRY-treated versus combined HDACIs and BRY treatment) were assessed by using a paired *t*-test. (*p < 0.05; **p < 0.005; ***p < 0.001).

## Additional Information

**How to cite this article**: Martínez-Bonet, M. *et al.* Synergistic Activation of Latent HIV-1 Expression by Novel Histone Deacetylase Inhibitors and Bryostatin-1. *Sci. Rep.* **5**, 16445; doi: 10.1038/srep16445 (2015).

## Supplementary Material

Supplementary Information

## Figures and Tables

**Table 1:** Dose-effect values of single drugs.

Cell type	Individual Drug	Dose-effect values			
		EC_50_	EC_75_	EC_90_	EC_95_
**J89GFP**	**BRY (μM)**	0.5 ± 0.18	2.02 ± 0.76	8.23 ± 3.41	21.48 ± 9.58
	**PNB (nM)**	49.85 ± 12.65	80.31 ± 25.32	130.36 ± 50.69	182.04 ± 80.92
	**RMD (nM)**	12.88 ± 2.88	20.17 ± 5.17	31.87 ± 10.13	43.72 ± 16.42
**THP89GFP**	**BRY (nM)**	17.3 ± 8.7	31.4 ± 9.8	62.6 ± 23.3	105.5 ± 55.6
	**PNB (nM)**	19.34 ± 6.43	28.87 ± 10.41	44.19 ± 18.99	60.02 ± 30.38
	**RMD (nM)**	10.55 ± 4.09	19.07 ± 6.9	35.83 ± 14.04	56.7 ± 27.87

The effective concentration (EC) for each drug after a 24-hour treatment of J89GFP and THP89GFP cells normalized to TNF-induced viral reactivation. Data are mean ± SD from at least six independent experiments performed in triplicate.

**Table 2:** Combination Index values (CI) of double combinations.

Double combination	Ratio	J89GFP				THP89GFP			
		EC_50_	EC_75_	EC_90_	EC_95_	EC_50_	EC_75_	EC_90_	EC_95_
BRY:PNB	2.5:2	0.56 ± 0.11 (+++)	0.48 ± 0.1 (+++)	0.42 ± 0.09 (+++)	0.38 ± 0.09 (+++)	1.16 ± 0.24 (−)	0.65 ± 0.11 (+++)	0.44 ± 0.12 (+++)	0.35 ± 0.11 (+++)
	5:2	0.41 ± 0.3 (+++)	0.34 ± 0.21 (+++)	0.33 ± 0.19 (+++)	0.34 ± 0.2 (+++)	1.27 ± 0.29 (−)	0.69 ± 0.32 (+++)	0.39 ± 0.2 (+++)	0.29 ± 0.15 (++++)
PNB:RMD	2:1	1.11 ± 0.1 (−)	0.92 ± 0.08 (±)	0.76 ± 0.06 (++)	0.67 ± 0.05 (+++)	2.05 ± 0.58 (−)	2.3 ± 0.37 (−)	2.71 ± 0.45 (−)	3.09 ± 0.79 (−)
BRY:RMD	2.5:1	0.47 ± 0.04 (+++)	0.26 ± 0.07 (++++)	0.16 ± 0.07 (++++)	0.11 ± 0.06 (++++)	0.88 ± 0.2 (+)	0.73 ± 0.29 (++)	0.64 ± 0.3 (+++)	0.63 ± 0.26 (+++)
	5:2	0.46 ± 0.04 (+++)	0.38 ± 0.05 (+++)	0.32 ± 0.05 (+++)	0.28 ± 0.05 (++++)	1.34 ± 0.42 (−)	0.87 ± 0.28 (+)	0.58 ± 0.18 (+++)	0.46 ± 0.15 (+++)

CI calculated at the EC_50_, EC_75_, and EC_90_. CI < 0.9 indicates synergism; 0.9 < CI < 1.1 indicates additive effects, and CI > 1.1 indicates antagonism (−). Synergy level: ± indicates additive effects; 0.85 < CI < 0.9 + (slight synergism); 0.7 < CI < 0.85 ++ (moderate synergism); 0.3 < CI < 0.7 +++ (synergism); CI < 0.1 < 0.3 ++++ (potent synergism). Compound range concentration: BRY (12.5–50 nM), PNB (5–40 nM) and RMD (2.5–20 nM). Each experiment was performed in duplicate. Data are represented as the mean ± SD of at least three independent experiments. **Abbreviations:** CI, combination index; EC_50_, 50% effective concentration; EC_75_, 75% effective concentration; EC_90_, 90% effective concentration; EC_95_, 95% effective concentration; SD, standard deviation; BRY, bryostatin-1; PNB, panobinostat; RMD, romidepsin.

**Table 3:** Combination Index values (CI) of triple combinations BRY:PNB:RMD.

Cell type	ratio 2.5:2:1				ratio 5:2:1			
	EC_50_	EC_75_	EC_90_	EC_95_	EC_50_	EC_75_	EC_90_	EC_95_
**J89GFP**	0.76 ± 0.16 (++)	0.57 ± 0.1 (+++)	0.42 ± 0.07 (+++)	0.35 ± 0.05 (+++)	0.47 ± 0.10 (+++)	0.35 ± 0.07 (+++)	0.26 ± 0.05 (++++)	0.22 ± 0.04 (++++)
**THP89GFP**	4.39 ± 2.02 (−)	1.92 ± 0.22 (−)	0.69 ± 0.11 (+++)	0.46 ± 0.1 (+++)	1.52 ± 0.62 (−)	0.84 ± 0.24 (++)	0.52 ± 0.09 (+++)	0.4 ± 0.06 (+++)

CI calculated at the EC_50_, EC_75_, EC90 and EC_95_. CI < 0.9 indicates synergism; 0.9 < CI < 1.1 indicates additive effects, and CI > 1.1 indicates antagonism (−). Synergy level: ± indicates additive effects; 0.85 < CI < 0.9 + (slight synergism); 0.7 < CI < 0.85 ++ (moderate synergism); 0.3 < CI < 0.7 +++ (synergism); CI < 0.1 < 0.3 ++++ (potent synergism). Compound range concentration: BRY (12.5–50 nM), PNB (5–40 nM) and RMD (2.5–20 nM). Each experiment was performed in duplicate. Data are represented as the mean ± SD of at least three independent experiments. **Abbreviations:** CI, combination index; EC_50_, 50% effective concentration; EC_75_, 75% effective concentration; EC_90_, 90% effective concentration; EC_95_, 95% effective concentration; SD, standard deviation; BRY, bryostatin-1; PNB, panobinostat; RMD, romidepsin.

**Table 4:** Dose-effect EC_75_ values of drugs alone or in combinations in THP89GFP cells.

	Individual Drug	Double combination					Triple combination	
		BRY:PNB (2.5:2)	BRY:PNB (5:2)	PNB:RMD (2:1)	BRY:RMD (2.5:1)	BRY:RMD (5:1)	BRY:PNB:RMD (2.5:2:1)	(5:2:1)
**BRY (nM)**	31.4 ± 9.8	21.1 ± 3.78*	26.67 ± 10.32		15.49 ± 3.96**	21.31 ± 3.49	37.18 ± 17.21	20.68 ± 3.95*
**PNB (nM)**	28.87 ± 10.41	16.88 ± 3.03*	10.67 ± 4.13***	23.81 ± 7.98			29.74 ± 13.75	8.27 ± 1.58***
**RMD (nM)**	19.07 ± 6.9			11.9 ± 3.99*	6.2 ± 1.59**	4.26 ± 0.7***	14.88 ± 6.89	4.14 ± 0.79***

The 75% effective reactivation concentration (EC_75_) values (nM ± SD) in latently infected THP89GFP cells of BRY, PNB, and RMD drugs alone and in combination at the indicated ratios 24 h post-treatment are shown. Data are represented as the mean ± SD of at least three independent experiments. Statistics were performed between the calculated EC_75_ values of the drug at single and combined treatments. ***p < 0.001; **p < 0.005; *p < 0.05.

## References

[b1] Battistini A. & Sgarbanti M. HIV-1 latency: an update of molecular mechanisms and therapeutic strategies. Viruses 6, 1715–1758, 10.3390/v6041715 (2014). 24736215 PMC4014718

[b2] Finzi D. *et al.* Identification of a reservoir for HIV-1 in patients on highly active antiretroviral therapy. Science 278, 1295–1300 (1997). 9360927 10.1126/science.278.5341.1295

[b3] Wong J. K. *et al.* Recovery of replication-competent HIV despite prolonged suppression of plasma viremia. Science 278, 1291–1295 (1997). 9360926 10.1126/science.278.5341.1291

[b4] Chun T. W. *et al.* Presence of an inducible HIV-1 latent reservoir during highly active antiretroviral therapy. Proceedings of the National Academy of Sciences of the United States of America 94, 13193–13197 (1997). 9371822 10.1073/pnas.94.24.13193 PMC24285

[b5] Stebbing J., Gazzard B. & Douek D. C. Where does HIV live? N Engl J Med 350, 1872–1880, 10.1056/NEJMra032395 (2004). 15115833

[b6] Carter C. C. *et al.* HIV-1 infects multipotent progenitor cells causing cell death and establishing latent cellular reservoirs. Nature medicine 16, 446–451, 10.1038/nm.2109 (2010). PMC2892382 20208541

[b7] Kulkosky J. & Bray S. HAART-persistent HIV-1 latent reservoirs: their origin, mechanisms of stability and potential strategies for eradication. Current HIV research 4, 199–208 (2006). 16611058 10.2174/157016206776055084

[b8] Margolis D. M. Histone deacetylase inhibitors and HIV latency. Curr Opin HIV AIDS 6, 25–29, 10.1097/COH.0b013e328341242d (2011). 21242890 PMC3079555

[b9] Shirakawa K., Chavez L., Hakre S., Calvanese V. & Verdin E. Reactivation of latent HIV by histone deacetylase inhibitors. Trends Microbiol 21, 277–285, 10.1016/j.tim.2013.02.005 (2013). 23517573 PMC3685471

[b10] Xing S. *et al.* Disulfiram reactivates latent HIV-1 in a Bcl-2-transduced primary CD4+ T cell model without inducing global T cell activation. Journal of virology 85, 6060–6064, 10.1128/JVI.02033-10 (2011). 21471244 PMC3126325

[b11] Doyon G., Zerbato J., Mellors J. W. & Sluis-Cremer N. Disulfiram reactivates latent HIV-1 expression through depletion of the phosphatase and tensin homolog. Aids 27, F7–F11, 10.1097/QAD.0b013e3283570620 (2013). 22739395

[b12] Zhu J. *et al.* Reactivation of latent HIV-1 by inhibition of BRD4. Cell Rep 2, 807–816, 10.1016/j.celrep.2012.09.008 (2012). 23041316 PMC3523124

[b13] Warrilow D., Gardner J., Darnell G. A., Suhrbier A. & Harrich D. HIV type 1 inhibition by protein kinase C modulatory compounds. AIDS research and human retroviruses 22, 854–864, 10.1089/aid.2006.22.854 (2006). 16989610

[b14] Kulkosky J. *et al.* Prostratin: activation of latent HIV-1 expression suggests a potential inductive adjuvant therapy for HAART. Blood 98, 3006–3015 (2001). 11698284 10.1182/blood.v98.10.3006

[b15] Hamer D. H. *et al.* Rational design of drugs that induce human immunodeficiency virus replication. Journal of virology 77, 10227–10236 (2003). 12970407 10.1128/JVI.77.19.10227-10236.2003 PMC228450

[b16] Mehla R. *et al.* Bryostatin modulates latent HIV-1 infection via PKC and AMPK signaling but inhibits acute infection in a receptor independent manner. PloS one 5, e11160, 10.1371/journal.pone.0011160 (2010). 20585398 PMC2886842

[b17] del Real G. *et al.* Statins inhibit HIV-1 infection by down-regulating Rho activity. The Journal of experimental medicine 200, 541–547, 10.1084/jem.20040061 (2004). 15314078 PMC2211926

[b18] Archin N. M. *et al.* Administration of vorinostat disrupts HIV-1 latency in patients on antiretroviral therapy. Nature 487, 482–485, 10.1038/nature11286 (2012). 22837004 PMC3704185

[b19] Rasmussen T. A., Tolstrup M., Winckelmann A., Ostergaard L. & Sogaard O. S. Eliminating the latent HIV reservoir by reactivation strategies: advancing to clinical trials. Hum Vaccin Immunother 9, 790–799, 10.4161/hv.23202 (2013). 23563519 PMC3903897

[b20] Yin H., Zhang Y., Zhou X. & Zhu H. Histonedeacetylase inhibitor Oxamflatin increase HIV-1 transcription by inducing histone modification in latently infected cells. Mol Biol Rep 38, 5071–5078, 10.1007/s11033-010-0653-6 (2011). 21181272

[b21] Victoriano A. F. *et al.* Novel histone deacetylase inhibitor NCH-51 activates latent HIV-1 gene expression. FEBS Lett 585, 1103–1111, 10.1016/j.febslet.2011.03.017 (2011). 21402072

[b22] Furumai R. *et al.* FK228 (depsipeptide) as a natural prodrug that inhibits class I histone deacetylases. Cancer Res 62, 4916–4921 (2002). 12208741

[b23] Zonder J. A. *et al.* A phase II trial of bryostatin 1 in the treatment of metastatic colorectal cancer. Clin Cancer Res 7, 38–42 (2001). 11205915

[b24] Bullen C. K., Laird G. M., Durand C. M., Siliciano J. D. & Siliciano R. F. New *ex vivo* approaches distinguish effective and ineffective single agents for reversing HIV-1 latency *in vivo*. Nature medicine 20, 425–429, 10.1038/nm.3489 (2014). PMC3981911 24658076

[b25] Reuse S. *et al.* Synergistic activation of HIV-1 expression by deacetylase inhibitors and prostratin: implications for treatment of latent infection. PLoS One 4, e6093, 10.1371/journal.pone.0006093 (2009). 19564922 PMC2699633

[b26] Perez M. *et al.* Bryostatin-1 synergizes with histone deacetylase inhibitors to reactivate HIV-1 from latency. Current HIV research 8, 418–429 (2010). 20636281 10.2174/157016210793499312

[b27] Chen L. F., Mu Y. & Greene W. C. Acetylation of RelA at discrete sites regulates distinct nuclear functions of NF-kappaB. EMBO J 21, 6539–6548 (2002). 12456660 10.1093/emboj/cdf660 PMC136963

[b28] Rasmussen T. A. *et al.* Comparison of HDAC inhibitors in clinical development: effect on HIV production in latently infected cells and T-cell activation. Hum Vaccin Immunother 9, 993–1001, 10.4161/hv.23800 (2013). 23370291 PMC3899169

[b29] International A. S. S. W. G. o. H. I. V. C. *et al.* Towards an HIV cure: a global scientific strategy. Nat Rev Immunol 12, 607–614, 10.1038/nri3262 (2012). 22814509 PMC3595991

[b30] Kutsch O., Benveniste E. N., Shaw G. M. & Levy D. N. Direct and quantitative single-cell analysis of human immunodeficiency virus type 1 reactivation from latency. Journal of virology 76, 8776–8786 (2002). 12163598 10.1128/JVI.76.17.8776-8786.2002 PMC136999

[b31] Chou T. C. Assessment of synergistic and antagonistic effects of chemotherapeutic agents *in vitro*. Contributions to gynecology and obstetrics 19, 91–107 (1994). 7995057

[b32] Chou T. C. Theoretical basis, experimental design, and computerized simulation of synergism and antagonism in drug combination studies. Pharmacological reviews 58, 621–681, 10.1124/pr.58.3.10 (2006). 16968952

[b33] Chou T. C. & Talalay P. Quantitative analysis of dose-effect relationships: the combined effects of multiple drugs or enzyme inhibitors. Adv Enzyme Regul 22, 27–55 (1984). 6382953 10.1016/0065-2571(84)90007-4

[b34] Spina C. A. *et al.* An in-depth comparison of latent HIV-1 reactivation in multiple cell model systems and resting CD4+ T cells from aviremic patients. PLoS Pathog 9, e1003834, 10.1371/journal.ppat.1003834 (2013). 24385908 PMC3873446

[b35] Lopez-Cabrera M. *et al.* Transcriptional regulation of the gene encoding the human C-type lectin leukocyte receptor AIM/CD69 and functional characterization of its tumor necrosis factor-alpha-responsive elements. J Biol Chem 270, 21545–21551 (1995). 7665567 10.1074/jbc.270.37.21545

[b36] Shan L. *et al.* Stimulation of HIV-1-specific cytolytic T lymphocytes facilitates elimination of latent viral reservoir after virus reactivation. Immunity 36, 491–501, 10.1016/j.immuni.2012.01.014 (2012). 22406268 PMC3501645

[b37] Jones R. B. *et al.* Histone deacetylase inhibitors impair the elimination of HIV-infected cells by cytotoxic T-lymphocytes. PLoS Pathog 10, e1004287, 10.1371/journal.ppat.1004287 (2014). 25122219 PMC4133386

[b38] Wightman F., Ellenberg P., Churchill M. & Lewin S. R. HDAC inhibitors in HIV. Immunol Cell Biol 90, 47–54, 10.1038/icb.2011.95 (2012). 22083528

[b39] Wei D. G. *et al.* Histone deacetylase inhibitor romidepsin induces HIV expression in CD4 T cells from patients on suppressive antiretroviral therapy at concentrations achieved by clinical dosing. PLoS Pathog 10, e1004071, 10.1371/journal.ppat.1004071 (2014). 24722454 PMC3983056

[b40] Van Lint C., Bouchat S. & Marcello A. HIV-1 transcription and latency: an update. Retrovirology 10, 67, 10.1186/1742-4690-10-67 (2013). 23803414 PMC3699421

[b41] Butera S. T., Perez V. L., Wu B. Y., Nabel G. J. & Folks T. M. Oscillation of the human immunodeficiency virus surface receptor is regulated by the state of viral activation in a CD4+ cell model of chronic infection. Journal of virology 65, 4645–4653 (1991). 1678437 10.1128/jvi.65.9.4645-4653.1991 PMC248919

[b42] Duh E. J., Maury W. J., Folks T. M., Fauci A. S. & Rabson A. B. Tumor necrosis factor alpha activates human immunodeficiency virus type 1 through induction of nuclear factor binding to the NF-kappa B sites in the long terminal repeat. Proceedings of the National Academy of Sciences of the United States of America 86, 5974–5978 (1989). 2762307 10.1073/pnas.86.15.5974 PMC297754

[b43] Folks T. M., Justement J., Kinter A., Dinarello C. A. & Fauci A. S. Cytokine-induced expression of HIV-1 in a chronically infected promonocyte cell line. Science 238, 800–802 (1987). 3313729 10.1126/science.3313729

[b44] Folks T. M. *et al.* Characterization of a promonocyte clone chronically infected with HIV and inducible by 13-phorbol-12-myristate acetate. J Immunol 140, 1117–1122 (1988). 2449497

[b45] Perez V. L. *et al.* An HIV-1-infected T cell clone defective in IL-2 production and Ca2+ mobilization after CD3 stimulation. J Immunol 147, 3145–3148 (1991). 1833465

[b46] Jordan A., Bisgrove D. & Verdin E. HIV reproducibly establishes a latent infection after acute infection of T cells *in vitro*. EMBO J 22, 1868–1877, 10.1093/emboj/cdg188 (2003). 12682019 PMC154479

[b47] Jordan A., Defechereux P. & Verdin E. The site of HIV-1 integration in the human genome determines basal transcriptional activity and response to Tat transactivation. EMBO J 20, 1726–1738, 10.1093/emboj/20.7.1726 (2001). 11285236 PMC145503

[b48] Prince H. M., Bishton M. J. & Johnstone R. W. Panobinostat (LBH589): a potent pan-deacetylase inhibitor with promising activity against hematologic and solid tumors. Future oncology 5, 601–612, 10.2217/fon.09.36 (2009). 19519200

[b49] Campbell G. R., Bruckman R. S., Chu Y. L. & Spector S. A. Autophagy Induction by Histone Deacetylase Inhibitors Inhibits HIV Type 1. J Biol Chem, 10.1074/jbc.M114.605428 (2014). PMC4335239 25540204

[b50] Clouse K. A. *et al.* Monokine regulation of human immunodeficiency virus-1 expression in a chronically infected human T cell clone. Journal of immunology 142, 431–438 (1989). 2463307

[b51] Folks T. M. *et al.* Tumor necrosis factor alpha induces expression of human immunodeficiency virus in a chronically infected T-cell clone. Proceedings of the National Academy of Sciences of the United States of America 86, 2365–2368 (1989). 2784570 10.1073/pnas.86.7.2365 PMC286913

[b52] Garcia-Merino I. *et al.* The Spanish HIV BioBank: a model of cooperative HIV research. Retrovirology 6, 27, 10.1186/1742-4690-6-27 (2009). 19272145 PMC2667474

