# Supplementary material for: Synergistic Activation of Latent HIV-1 Expression by Novel Histone Deacetylase Inhibitors and Bryostatin-1
Source: Sci Rep. 2015 Nov 13;5:16445. doi: 10.1038/srep16445 (PMC4643323; doi:10.1038/srep16445)
Supplement: Supplementary Information [file srep16445-s1.pdf]

Synergistic Activation of Latent HIV-1 Expression by Novel Histone Deacetylase Inhibitors and Bryostatin-1

Marta Martínez-Bonet [a], Maria Isabel Clemente [a,b], Maria Jesús Serramía [a], Eduardo Muñoz [c], Santiago Moreno [d], Maria Ángeles Muñoz-Fernández [a,*]

[a] Laboratorio de InmunoBiología Molecular, Hospital General Universitario Gregorio Marañón; Instituto de Investigación Sanitaria Gregorio Marañón (IISGM), and Networking Research Center on Bioengineering, Biomaterials and Nanomedicine (CIBER-BBN); C/ Dr. Esquerdo 46, 28007 Madrid, Spain

[b] Unidad de cultivos celulares, Instituto de Investigación Sanitaria Gregorio Marañón (IISGM), C/ Dr. Esquerdo 46, 28007 Madrid, Spain

[c] Departamento de Inmunología, Facultad de Medicina, Universidad de Córdoba, Avda. Menéndez Pidal, s/n. 14004, Córdoba, Spain

[d] Servicio de Enfermedades Infecciosas, Hospital Universitario Ramón y Cajal and IRYCIS, Madrid, Spain

* Corresponding author. E-mail address: mmunoz.hgugm@gmail.com. Tel.: +34915868565; fax number: +34915868018

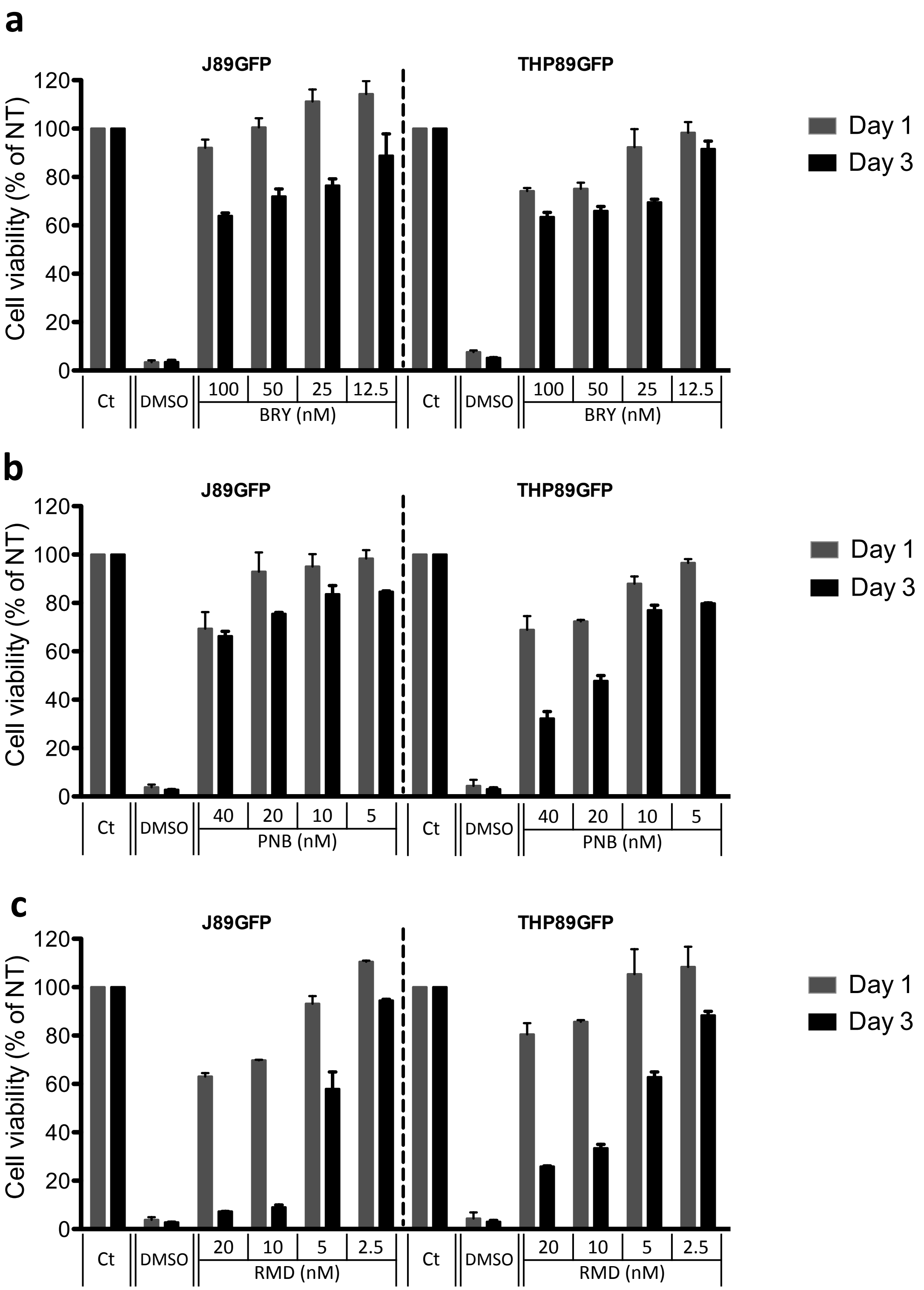

Supplementary Fig. S1. Cell viability of J89GFP and THP89GFP cells after single drug treatment. Cells were treated for 1 or 3 days (grey and black bars, respectively) with a concentration range of BRY **(a)**, PNB **(b)** or RMD **(c)**. Cell survival was determined using the MTT cytotoxicity assay. Results are normalized to control vehicle-treated cells (Ct) and represent the mean + SEM of three independent experiments performed in triplicate.

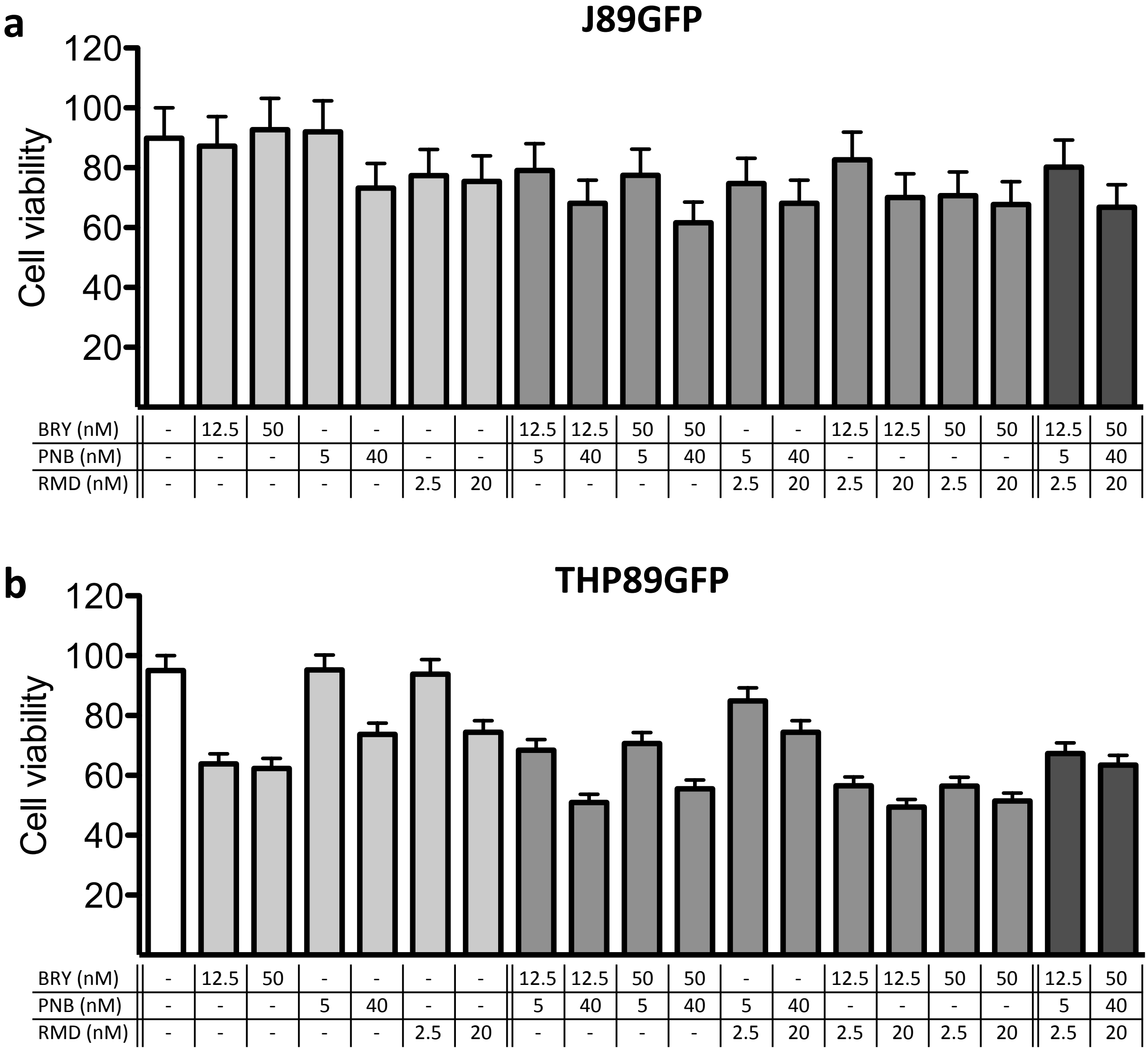

Supplementary Fig. S2. Cell viability of J89GFP and THP89GFP cells after treatment with drug combinations. Cells were treated for 24 hours with the indicated concentrations of BRY, PNB or RMD alone or in combination and cell viability was determined by FACS analysis of 7AAD staining. White bar corresponds to control vehicle-treated cells (Ct), light grey bars correspond to single drug treatment, grey bars correspond to double combinations, and dark grey bars correspond to triple combinations. Results represent the mean + SEM of three independent experiments performed.

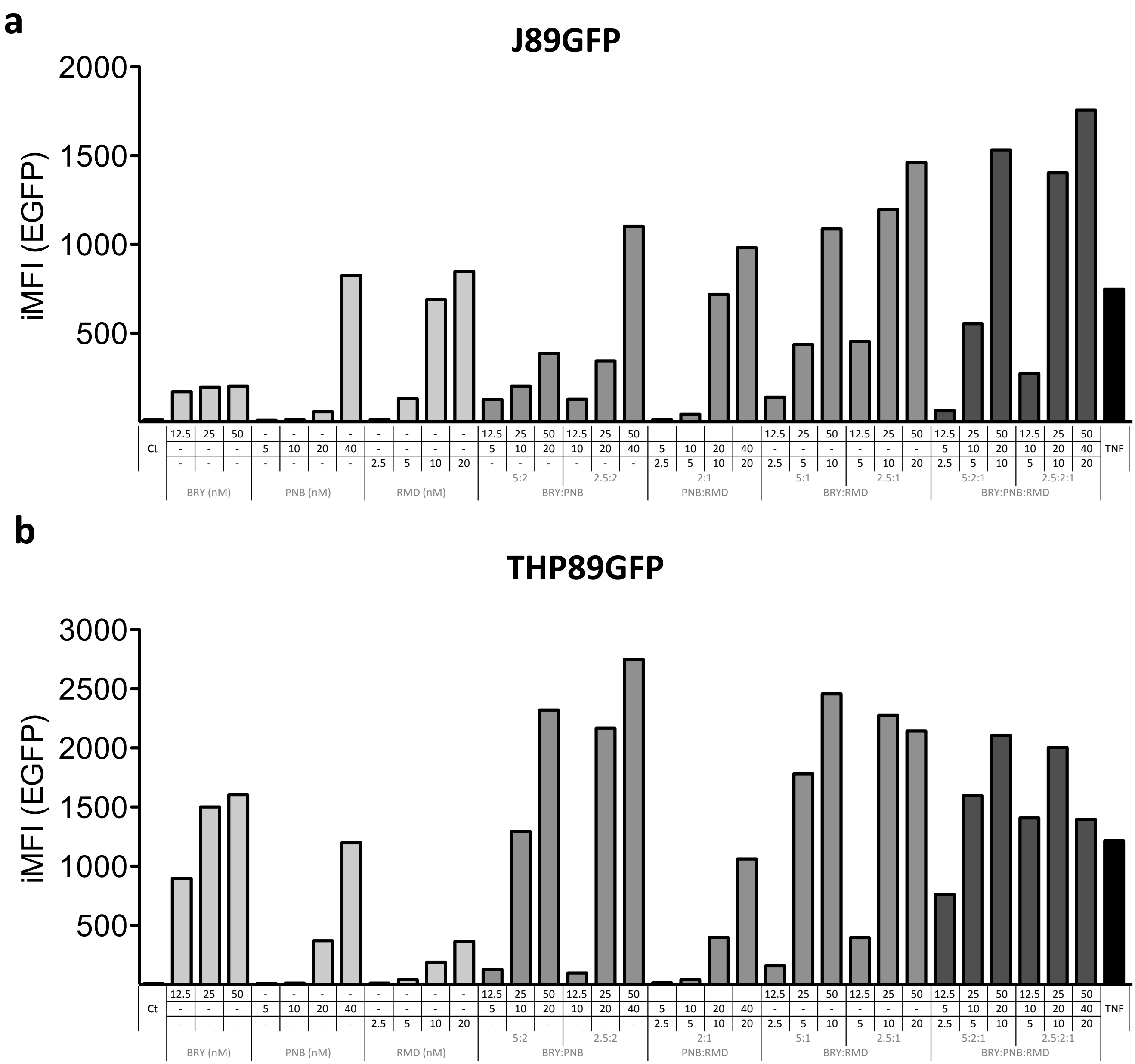

Supplementary Fig. S3. Reactivation-effect of drugs alone or in double combinations. J89GFP **(a)** and THP89GFP **(b)** cells were treated with BRY, PNB and RMD at the indicated concentrations, alone or in combination. After 24 hours, HIV-1 reactivation was analysed by flow cytometry as EGFP expression (iMFI). White bar corresponds to control vehicle-treated cells (Ct), light grey bars correspond to single drug treatment, grey bars correspond to double combinations, dark grey bars correspond to triple combinations and black bar (TNF) corresponds to TNF-α treatment. Results represent the arithmetic mean + SEM of at least three independent experiments.

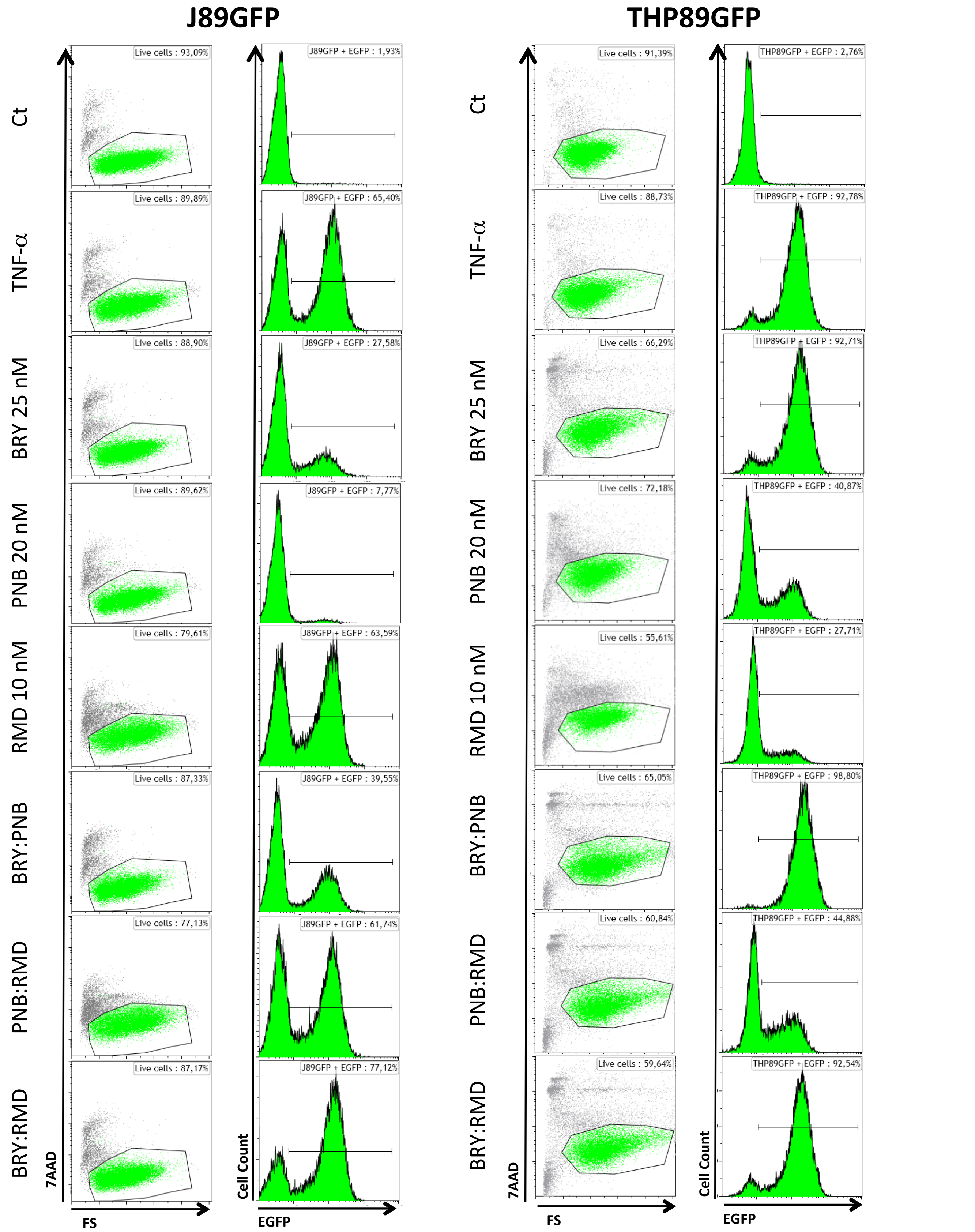

Supplementary Fig. S4. Representative flow profiles of J89GFP and THP89GFP cell viability and viral reactivation after drug treatment. Cells were treated with BRY, PNB and RMD at the indicated concentrations, alone or in combination. Representative flow profiles of 7AAD staining and EGFP expression are shown for each condition. Percentage of live cells and EGFP positive cells are indicated in each flow chart. FS, forward scatter.

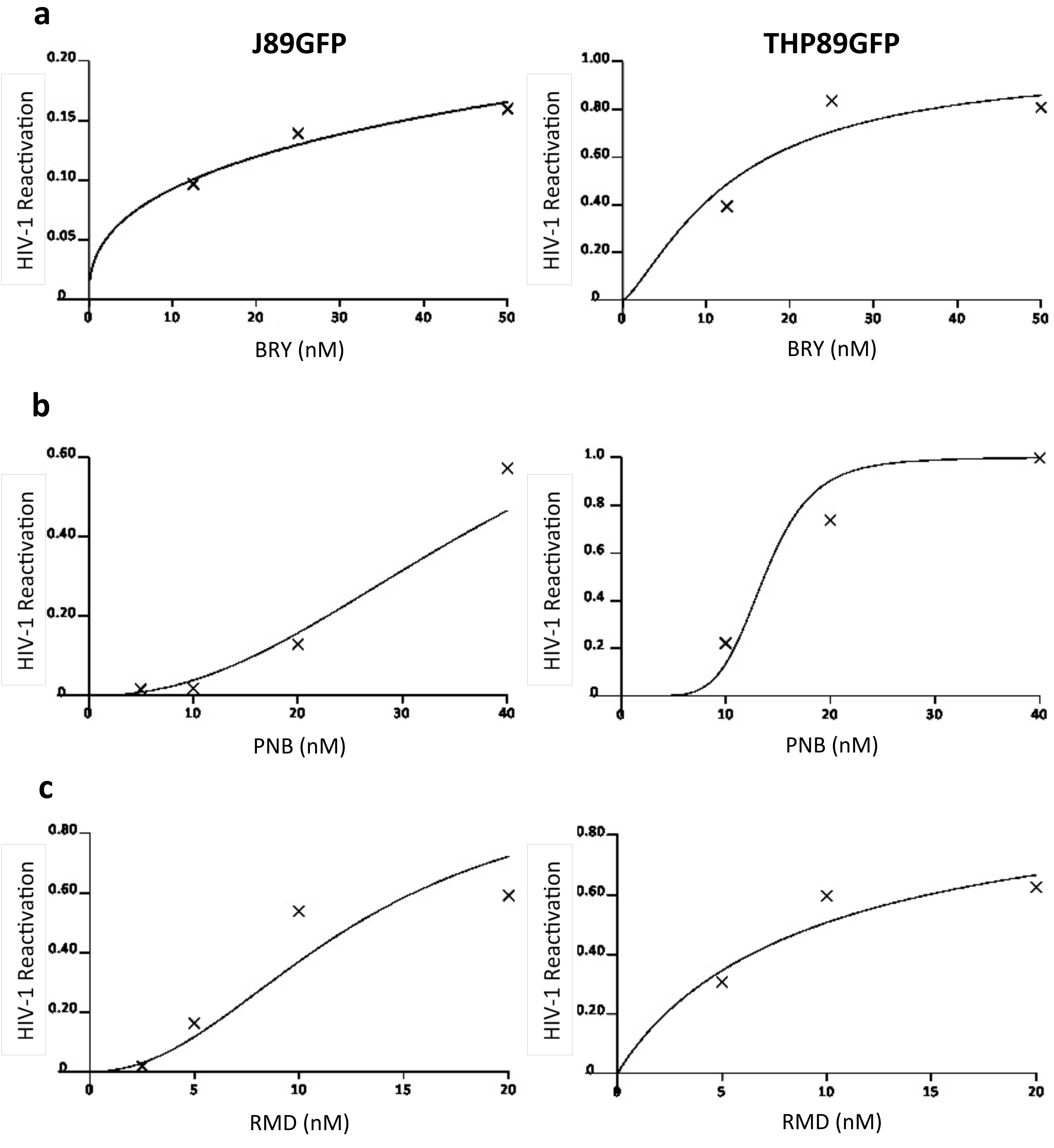

Supplementary Fig. S5. Dose-effect curves of single drugs. Latently infected J89GFP (left panels) and THP89GFP (right panels) cells were incubated in the presence of BRY **(a)**, PNB **(b)** or RMD **(c)**. Dose response of HIV reactivation was determined by the quantification of EGFP expression after a 24-hours treatment. Results from a representative experiment are shown.

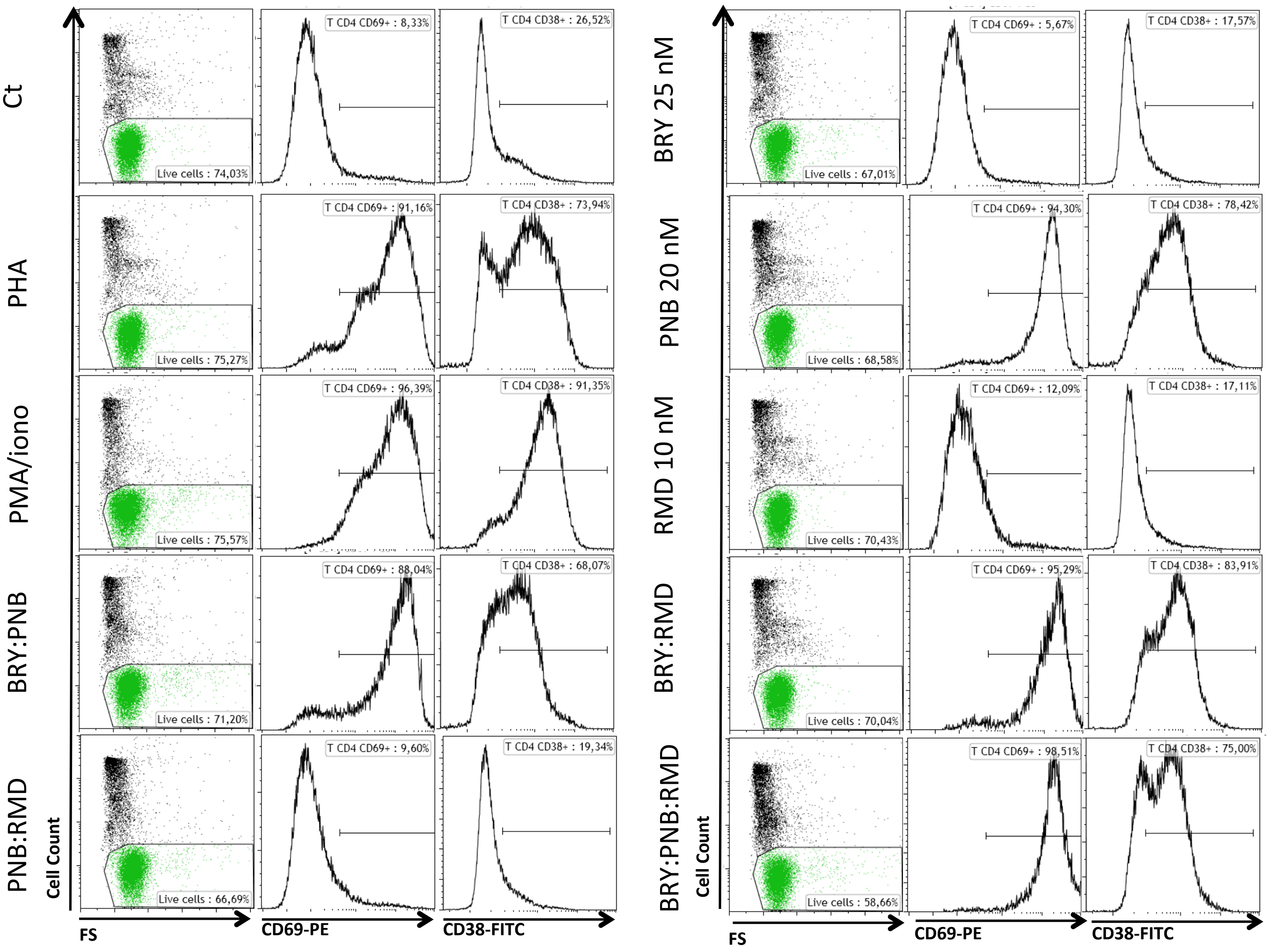

Supplementary Fig. S6. Representative flow profiles of primary CD4 T cell viability and activation levels after drug treatment. Purified CD4 T cells from healthy subjects were treated with the indicated concentrations of BRY, PNB, RMD or with PHA or PMA/ionomycin. Representative flow profiles of 7AAD staining and the activation markers CD38 and CD69 in viable CD4 T cells after 1 day of treatment are shown. Percentage of live cells, CD69 and CD38 positive cells are indicated in each flow chart. FS, forward scatter.

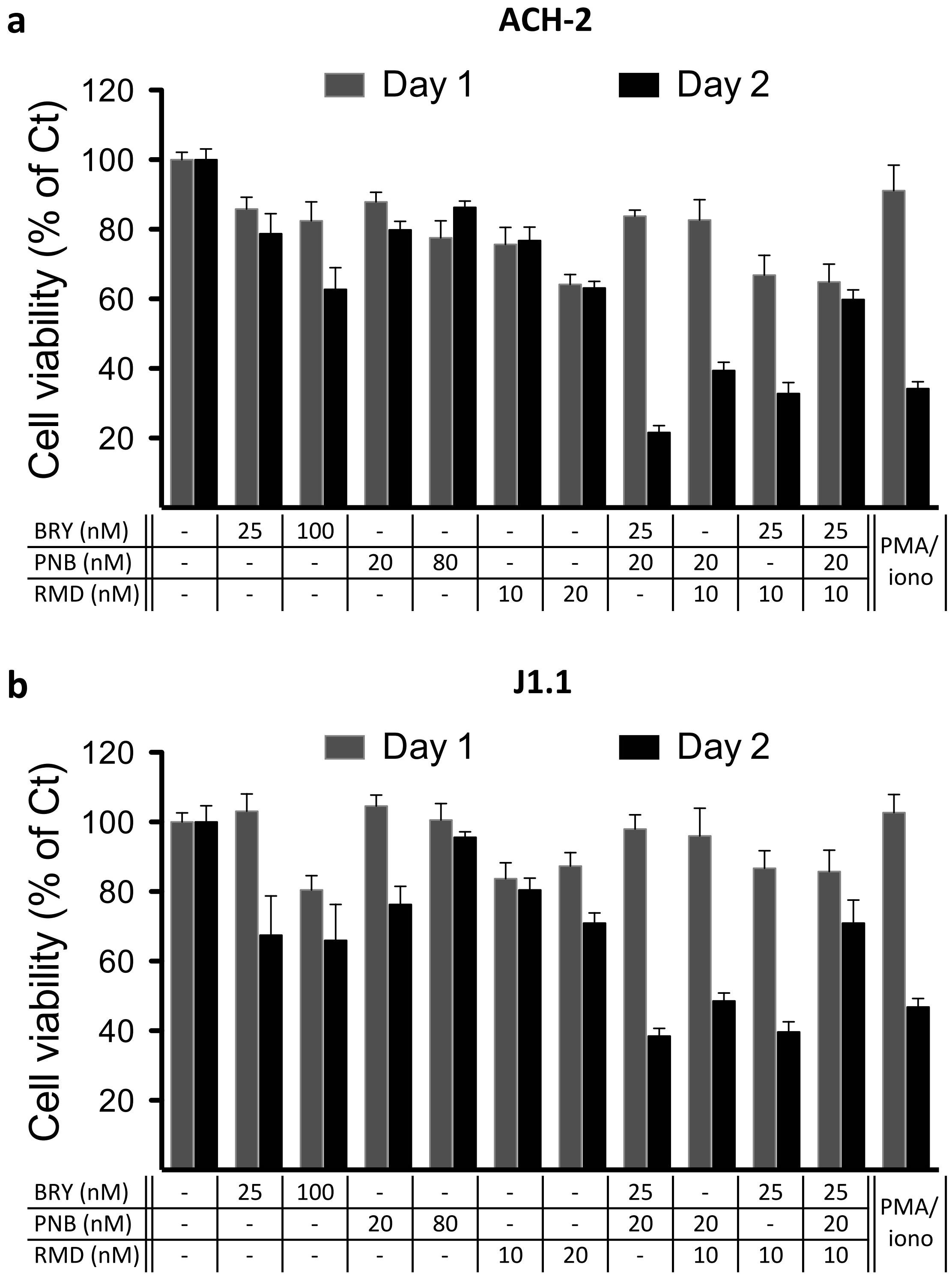

Supplementary Fig. S7. Cell viability of ACH-2 and J1.1 cells after single or combined drug treatment. ACH-2 **(a)** and J1.1 **(b)** cells were treated for 1 or 2 days (grey and black bars, respectively) with the indicated concentrations of BRY, PNB, RMD or with PMA/ionomycin. Cell survival was determined using the MTT cytotoxicity assay. Results are normalized to control vehicle-treated cells (Ct) and represent the mean + SEM of three independent experiments performed in triplicate.

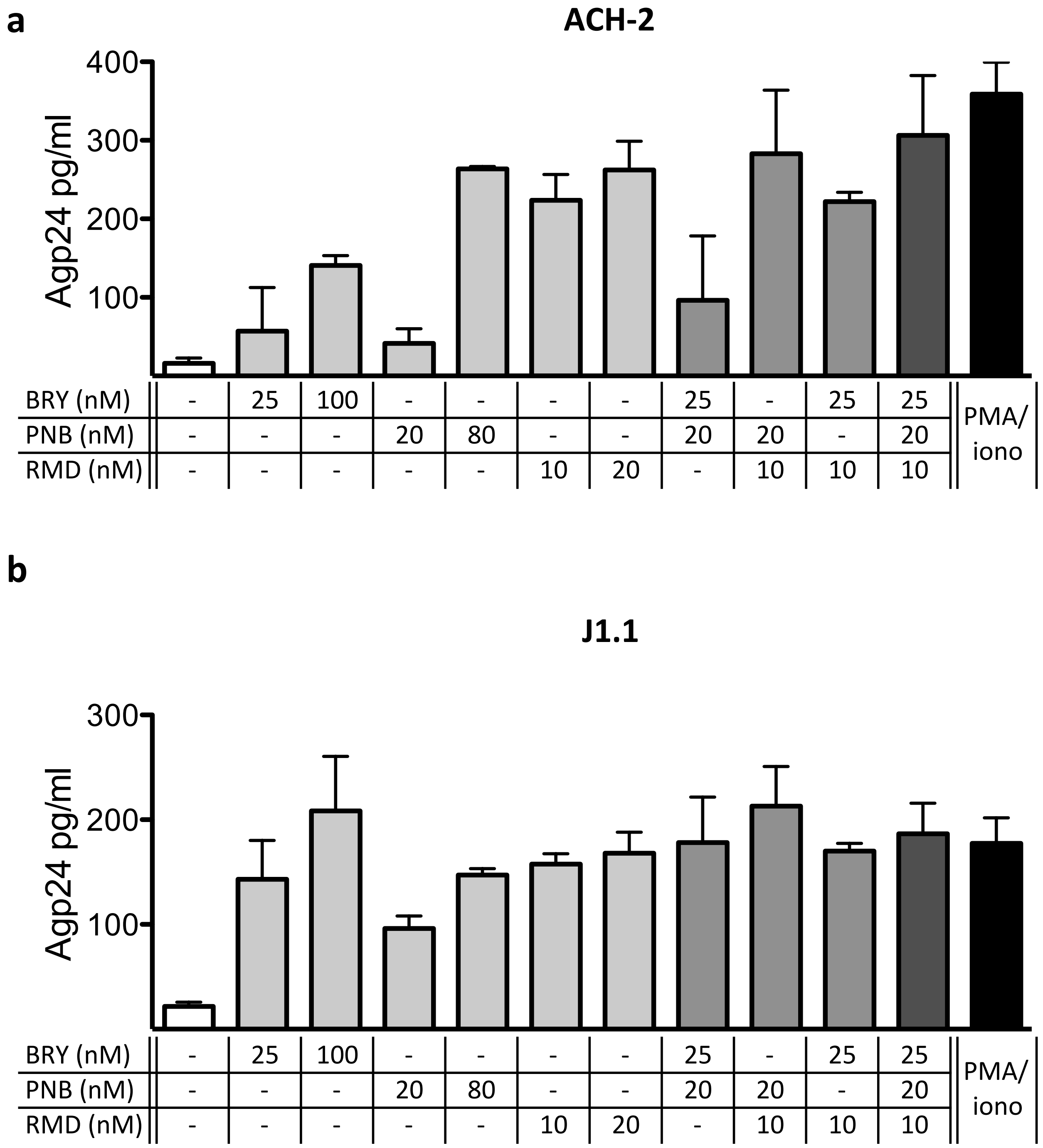

Supplementary Fig. S8. HIV-1 reactivation in ACH-2 and J1.1 cells after single or combined drug treatment. ACH-2 **(a)** and J1.1 **(b)** cells were treated for 1 day with the indicated concentrations of BRY, PNB, RMD or with PMA/ionomycin. HIV-1 reactivation was analysed as Agp24 release measured by ELISA. White bar corresponds to control vehicle-treated cells (Ct), light grey bars correspond to single drug treatment, grey bars correspond to double combinations, dark grey bar corresponds to triple combination and black bar correspond to the positive control stimuli PMA/ionomycin.
